# Quantum suppression of antihydrogen formation in positronium-antiproton scattering

**DOI:** 10.1038/s41467-017-01721-y

**Published:** 2017-11-16

**Authors:** A. S. Kadyrov, I. Bray, M. Charlton, I. I. Fabrikant

**Affiliations:** 1 Curtin Institute for Computation and Department of Physics, Astronomy and Medical Radiation Science, Curtin University, GPO Box U1987, Perth, WA 6845 Australia; 2 Department of Physics, College of Science, Swansea University, Swansea, SA2 8PP UK; 3 Department of Physics and Astronomy, University of Nebraska, Lincoln, NE 68588-0299 USA

## Abstract

The interaction of antiprotons with low-energy positronium atoms is a fundamental three-body problem whose significance is its utility for formation of antihydrogen. Particular importance resides in understanding processes involving excited positronium states. Until recently such studies were performed using classical techniques. However, they become inapplicable in the low-energy domain. Here we report the results of comprehensive quantum calculations, which include initial excited positronium states with principal quantum numbers up to *n*_i_ = 5. Contrary to expectation from earlier work, there are only muted increases in the cross-sections for antihydrogen formation for *n*_i_ > 3. We interpret this in terms of quantum suppression of the reaction at higher angular momenta. Furthermore, the cross-sections for elastic scattering are around two orders of magnitude higher, which we attribute to the degeneracy of the positronium states. We outline some experimental consequences of our results.

## Abstract (web summary)

The intriguing behaviour of positronium scattering has its role in antimatter studies. Here the authors have predicted the quantum suppression of the cross-section for antihydrogen formation in positronium scattering with antiprotons, by including the excited states and using convergent close-coupling calculations.

## Introduction

It was suggested some time ago^1–3^ that it might be advantageous to form antihydrogen by $\bar p$-Ps scattering via the process,

1 $${\mathrm{Ps}}\left( {n_{\rm i},l_{\rm i}} \right) + \bar p \to {\bar{\mathrm H}}\left( {n{\prime},l{\prime}} \right) + e^ - ,$$

where the Ps initial state is characterised by principal and orbital angular momentum quantum numbers *n*_i_ and *l*_i_, respectively, with *n*′ and *l*′ being the corresponding values for ${\bar{\mathrm H}}$. The community with an active interest in this field has grown considerably in recent years, and it is now predominantly experimental and includes some hundreds of scientists associated with work at $\bar p$ facilities of the European Organization for Nuclear Research (CERN). Their work is concerned with the total antihydrogen-formation cross-sections, $\sigma _{{\bar{\mathrm H}}}$, for different Ps principal quantum numbers, and it is this that motivates calculational effort. Due to charge conjugation symmetry, the problem is identical to that of proton-Ps scattering. However, it was not until relatively recently that accurate theoretical cross-sections for reaction 1 were forthcoming for Ps in other than the ground state, and at the low-kinetic energies (sub-eV) of current experimental interest^4,5^. That work found increases of several orders of magnitude in $\sigma _{{\bar{\mathrm H}}}$ when *n*_i_ was raised from 1 to 3, with the cross-sections for the excited states displaying a characteristic 1/*E* dependence at low Ps kinetic energies, *E*. These advances were possible due to progress with the two-centre convergent close-coupling (CCC) method^6,7^, a technique which is well suited to systems involving charge exchange.

An extension of the CCC studies^8^ discovered that cross-sections for elastic and quasi-elastic scattering

2 $${\mathrm{Ps}}\left( {n_{\rm i},l_{\rm i}} \right) + \bar p \to {\mathrm{Ps}}\left( {n_{\rm f},l_{\rm f}} \right) + \bar p,$$

were up to three orders of magnitude larger than those for reaction 1. (Here elastic scattering corresponds to the case when i = f, whilst quasi-elastic, or *l*-changing, scattering occurs when *n*_i_ = *n*_f_ with *l*_i_ ≠ *l*_f_.) Calculations^9^ also confirmed the existence of the dipole-supported resonances^10–15^ in collisions involving excited Ps or excited (anti)hydrogen.

Several prominent groupings at CERN aim to utilise reaction 1 to form antihydrogen in an effort to measure spectroscopic and gravitational properties of antimatter. The gravitational behaviour of antihydrogen at rest (GBAR) team^16–18^ intends to use a further interaction of the nascent ${\bar{\mathrm H}}$ with a Ps target to form the antihydrogen positive ion, which will be caught and cooled before being photo-ionised to allow the remnant ultra-cold ${\bar{\mathrm H}}$ to fall in the Earth’s gravitational field^19^. The antihydrogen experiment: gravity, interferometry, spectroscopy (AEgIS) collaboration^20,21^ will excite Ps atoms to high lying, so-called Rydberg, states to produce, by Stark acceleration^22,23^, a ${\bar{\mathrm H}}$ beam for deflectometry studies. The antihydrogen trap (ATRAP) experiment has observed reaction 1 following the production of highly excited (corresponding to *n*_i_ around 26) Ps using a secondary charge transfer reaction involving *e* ^+^ interactions with laser excited Cs atoms^24,25^. Recent activities of this collaboration, in which record numbers of cold *e* ^+^ have been stored^26^ to produce dramatically enhanced yields of excited state Ps^27^, suggest a renewed interest in this reaction.

The atomic spectroscopy and collisions using slow antiprotons (ASACUSA) programme^28^ aims to use a beam of antihydrogen to probe the ground state hyperfine transition. Currently the group exploits the three-body recombination scheme as $\bar p + e^ + + e^ + \to {\bar{\mathrm H}} + e^ +$, however it is well known that under the conditions needed for useful yields, this reaction produces a slew of states with binding energies in the meV region, and typically not in a beam-like geometry (e.g., refs ^29,30^). This implies that the weak downstream ${\bar{\mathrm H}}$ signals reported by this collaboration^28,31^ contain small ground state fractions. The simulation work conducted by subsets of the collaboration which aim to find optimum $\bar p$-*e* ^+^ conditions for ground state ${\bar{\mathrm H}}$ yields (see, for instance^32,33^) do not seem to provide definitive guidance.

There is no doubt that the Ps-$\bar p$ reaction is cleaner, producing more well-defined states, and with directional properties dominated by those of the colliding antiprotons. In general, experimentation with excited Ps atoms is an enterprise of growing significance, and several groups have developed the capability to efficiently produce a wide range of excited states^34–40^. These studies have benefited from the availability of robust sources of Ps in vacuum with kinetic energies below 100 meV (e.g., refs. ^37,41–44^). Indeed, it has recently been suggested^45^ that collisions of excited state Ps with cold ions may be used to produce a wide range of species of cold (and possibly trapped) atoms, and in particular elements that have so far proven impossible to cool directly.

Given the experimental progress and ambition, the importance of accurate input from theory is paramount in allowing rates of reaction to be assessed and expected measurement precision to be gauged. A recent effort in this direction is the work of Krasnický et al.^46^ who have considered Rydberg Ps-$\bar p$ collisions using a classical-trajectory Monte Carlo (CTMC) technique. As expected from such a treatment^2,24^, it was found that the antihydrogen-formation cross-sections scaled as $n_i^4$ across a range of kinetic energies below 1 eV with values as high as 10^−12^ m^2^ for *n*_i_ = 50. This scaling is very well known from the classical theory of Rydberg atom collisions with charged particles^47^. The calculation reproduced the 1/*E* dependence found from the CCC and threshold theory^9^ and also achieved reasonable accord with the absolute values for $\sigma _{{\bar{\mathrm H}}}$ from the CCC study where the two overlapped for *n*_i_ = 3.

In this work, motivated by experimental challenges and progress, we extend the previous studies by considering Ps principal quantum numbers up to *n*_i_ = 5. In doing so we elucidate an effect underlying the interaction of excited state Ps with ionic species.

## Results

### CCC calculations

**Fig. 1:**
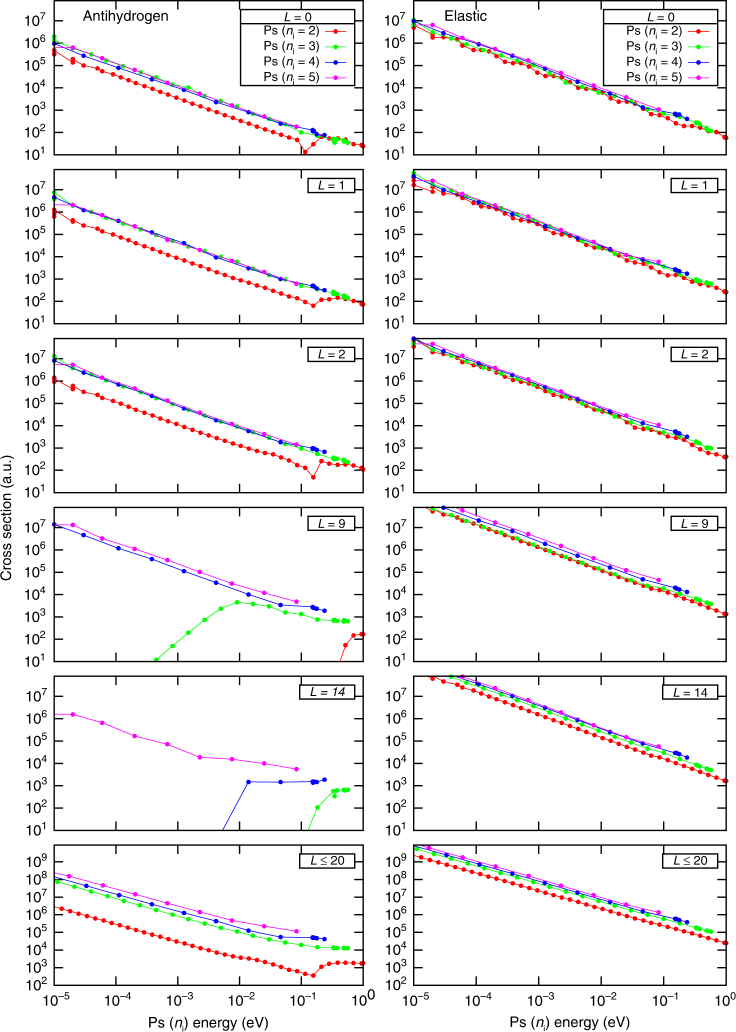
Antihydrogen-formation and elastic-scattering cross-sections. Cross-sections for Ps(*n*_i_, *l*_i_) + $\bar p$ reactions leading to ${\bar{\mathrm H}}$ formation (left) and elastic scattering (right) in atomic units (a.u.), where 1 a.u. is ~2.7 × 10^−21^ m^2^. The data are summed across the angular momentum manifold (0 ≤ *l*_i_ ≤ *n*_i_ − 1) for each specified *n*_i_. The ${\bar{\mathrm H}}\,\left( {n{\prime},l{\prime}} \right)$ formation cross-sections are summed over all possible cross-sections for which the reaction is exothermic $\left( {\varepsilon {_{n_{\rm{i}}}^{{\mathrm{Ps}}}  >\varepsilon _{n{\prime}}^{{\bar{\mathrm H}}}}} \right)$. The behaviour of the partial waves *L* = 0, 1, 2, 9, 14, and summed to *L* = 20 is shown. Note that only the summed cross-section for ${\bar{\mathrm H}}$ formation is convergent for *L* ≤ 20. The elastic cross-section formally increases to infinity with increasing partial waves due to the degeneracy of the Ps levels for *n*_i_ ≥ 2. See text for details

In order to further understand reaction 1, CCC calculations have been extended up to *n*_i_ = 5 with *n*′ ≤ 7 (Methods section). In addition, we have extended the threshold theory for elastic and quasi-elastic scattering^9^ to higher *n*_i_ and found that corresponding partial cross-sections are in very good agreement with the CCC calculations. Data for reactions 1 and 2 for *n*_i_ = 2–5 are presented in Fig. 1. The trends with increasing partial wave number *L* are illustrated with examples for *L* = 0, 1, 2, 9, and 14 plus the cross-sections summed up to *L* = 20. For antihydrogen formation the partial wave summed cross-section is fully converged with *L*. The data are given aggregated across the *l*-manifold at each *n*_i_. Although such cross-sections are artificial constructs, since experimentally selected individual Ps(*n*_i_, *l*_i_) states will be produced, presentation in this manner allows us to vividly draw attention to our main discoveries.

It is clear from Fig. 1 that the dramatic increase in cross-section for charge transfer when *n*_i_ is increased from 2 to 3 (which is also the case when comparing the data for *n*_i_ = 1 and 2^4,5^) is absent for the higher values of *n*_i_. The data for the lower partial waves display a remarkable consistency—the cross-sections for all processes are more-or-less identical for *n*_i_ = 3–5, and differences are only evident in the charge transfer process with the inclusion of higher *L* waves. Here, in the Ps kinetic energy region where the data for all *n*_i_ behave as 1/*E*, there is a factor of around 3 between the $\sigma _{{\bar{\mathrm H}}}$ for *n*_i_ = 5 when compared to *n*_i_ = 3. This is to be contrasted with the approximately factor of 30 jump between *n*_i_ = 2 and 3. As discussed below (Methods section) higher partial waves should be included as *n*_i_ rises which requires a more complete description of the collision, and it is these partial waves that produce the upward trend of $\sigma _{{\bar{\mathrm H}}}$ with *n*_i_, though it is not as dramatic as in the classical simulations.

A key feature of the CCC method is its unitarity for each *L*, and that convergence with *L* needs to be obtained. Presently, *L* ≤ 20 is sufficient for convergence in the ${\bar{\mathrm H}}$-formation cross-sections. However, because the elastic channel includes the quasi-elastic transitions, this cross-section diverges with increasing *L* unless relativistic splitting between degenerate levels is taken into account^9^. The unitarity aspect of the quantum mechanical approach to the problem is critically important here: it guarantees probability conservation between all possible reaction channels. Given the magnitude of the (quasi) elastic channels for each partial wave, the much smaller charge exchange process can only be accurately calculated with coupling between all the channels included.

### Threshold theory

To interpret the results presented above we note that the interaction between an excited Ps and a $\bar p$ decays as 1/*r* ^2^ at large *r*, where *r* is the Ps-$\bar p$ distance. This happens because degenerate states of Ps(*n*_i_, *l*_i_), *l*_i_ = 0, 1, ..., *n*_i_−1 in the presence of a point charge are coupled by the dipole interaction. (In particular, this causes the linear Stark effect in hydrogen-like atoms in excited states.) Classically, the non-zero dipole moment of the excited hydrogen-like system is due to closed Kepler orbits in the Coulomb field, therefore the same effective interaction occurs in classical simulations. Classical scattering cross-sections in the 1/*r* ^2^ potential are proportional to 1/*E* for any impact parameter^48^, and the classical transition probability remains finite at zero energy. According to the CTMC simulations^46^ this probability is non-negligible for all impact parameters up to those corresponding to the size of the Ps atom, which is proportional to $n_{\rm i}^2$. Therefore the classical charge transfer cross-section scales as $n_{\rm i}^4{\mathrm{/}}E$.

In quantum mechanics, although we deal with the same dipole interaction, the approach is different because at low-collision energies we must take into account the wavelike nature of Ps, and as such the behaviour is governed by the Wigner threshold law^49^. In the absence of a long-range interaction in the initial state, such as for Ps in its ground state, the Wigner analysis (Methods section) tells us that the cross-sections for an exothermic reaction have an *E* ^−1/2^ dependence, also in accord with CCC and threshold analyses^4,5,9^. However, for the excited states of Ps, the aforementioned long-range effective dipole interaction modifies the low-energy Ps-$\bar p$ behaviour. This happens because the dipole interaction competes with the centrifugal potential, and as a result the threshold exponent is modified^10,12^. If the dipole interaction is relatively weak compared to the centrifugal barrier, it can be shown (Methods section) that the partial charge transfer cross-section, *σ*_*L*_, has the form *E* ^*λ*−1/2^, where *λ* is a parameter which grows with *L*. This means that *σ*_*L*_ decreases exponentially with increasing *L* as E tends to zero: such behaviour can be called quantum suppression. On the other hand, if the dipole interaction dominates, the low-energy behaviour of *σ*_*L*_ is given by 1/*E*.

Below we assign a value *L*_b_ which marks the angular momentum boundary between the region where the partial cross-sections exhibit either the 1/*E* (dipole dominant), or the *E* ^*λ*−1/2^ (centrifugal barrier dominant) behaviour. To find *L*_b_ for each principal quantum number *n*_i_, we have extended the threshold theory to higher *n*_i_. The mathematical problem is reduced to the analysis of eigenvalues of a matrix which combines dipole interaction terms and centrifugal terms. Although this analysis is very simple computationally, it leads to a remarkable feature never noticed in the past and shown in Table 1: *L*_b_ grows linearly with *n*_i_. Thus, the *L* = 9 cross-section should be suppressed for *n*_i_ ≤ 3, and for *L* = 14 this should be the case for *n*_i_ ≤ 4. This is clearly confirmed in the Figure.

**Table 1:** Threshold theory boundary parameters

*n*_i_	*L*_b_	*μ*
3	8	2.35
4	12	3.62
5	15	1.21
6	19	2.93
7	23	4.45
8	27	5.91

Initial Ps principal quantum number *n*_i_, boundary value *L*_b_ and exponent *μ* such that for *L* = *L*_b_ + 1 the partial charge transfer cross-section *σ*_*L*_ is suppressed at low energies *E* due to near threshold behaviour *σ*_*L*_ ∝ *E* ^*μ*^

From the point of view of an impact parameter description, this means that the cutoff impact parameter *b*_max_ = *L*_b_/*k*, (where *k* is the initial Ps wave number) grows linearly with *n*_i_ rather than quadratically as in CTMC calculations. Accordingly, the quantum-mechanical cross-section grows as $n_{\rm i}^2$ rather than as $n_{\rm i}^4$, which is characteristic of the CTMC calculations. Note that this general trend is valid for sufficiently high *n*_i_. Nevertheless, the scaling is in reasonable accord with the ratio of the cross-sections for the *n*_i_ = 5 and *n*_i_ = 3 states from the CCC calculation. We note further that the results of the threshold theory are valid if the Ps wavelength is large compared to its size. This gives an upper limit for *n*_i_ for which the $n_{\rm i}^2$ scaling applies. For example, at *E* = 10^−3^ eV the scaling is valid if *n*_i_ < 15. It is possible that for much higher *n*_i_ the classical $n_{\rm i}^4$ scaling would be restored.

## Discussion

We conclude that, due to quantum-mechanical effects, the growth of the charge-transfer cross-section with *n*_i_ is much slower than in the classical calculations at moderate values of *n*_i_ where the threshold theory predicts $n_{\rm i}^2$ scaling. This arises primarily due to the limited number of partial waves contributing to the process. Although CCC calculations were performed only up to *n*_i_ = 5, the threshold theory analysis confirms this trend and allows us to extrapolate this prediction to higher *n*_i_. More generally, the outcome of $\bar p$-Ps scattering is, for excited states, dominated by the long-range interaction due to the degeneracy of the Ps energy levels, which in turn leads to the importance of the elastic and quasi-elastic channels of reaction 2.

Our work has elucidated the basic physics underlying the interaction of excited Ps atoms with protons and antiprotons. In the framework of non-relativistic theory, in which the various angular momentum states within a given *n*_i_ manifold are degenerate in energy, we find that quantum effects dramatically suppress the increase of the charge transfer cross-section, $\sigma _{{\bar{\mathrm H}}}$, in sharp contrast to expectations from Bohr-like and classical theories. This effect is independent of the charged species with which the Ps interacts, since it is caused by the inherent properties of interactions with Ps in excited states in the absence of effects (such as applied fields) that can lift the level degeneracy. If the trend in $\sigma _{{\bar{\mathrm H}}}$ persists at high *n*_i_, then the implications for current experimental efforts which aim to exploit efficient charge transfer from excited state Ps could be severe, especially in the light of heating due to large momentum transfer^8^.

Unfortunately, there is little experimental information with which to compare our results. A sole point of reference is the aforementioned work of the ATRAP collaboration that detected ${\bar{\mathrm H}}$ formed via reaction 1 in an apparatus employing a 5.3 T magnetic field^25^. Details are limited, but charge exchange cross-sections in the region of 10^−13^ m^2^ (around 3 × 10^7^ a.u.) for the formation of meV Ps were deduced^50^. Given the uncertainty, this value does not seem unreasonable when compared to the scale of those in Fig. 1.

We note that most antihydrogen experiments (e.g., refs ^28,51–54^) involve applied fields which will lift the energy degeneracy, and certainly at high *n*_i_ the behaviour of the Ps level structure will be complex. Unfortunately, such a Ps-field system is not readily amenable to theory to yield accurate scattering cross-sections. As a result, and given the scarcity of information available, experimental investigations of the interactions of simple ionic species with excited state Ps are of the utmost importance and are probably the only means of assessing the future viability of several ongoing programmes and forthcoming initiatives.

## Methods

### The CCC approach

The problem to be solved as expressed by (1) and (2) is the same, by symmetry, if the $\bar p$ is replaced by *p* and ${\bar{\mathrm H}}$ by H. In the two-centre CCC approach^6^ the total wave function of the system is expanded in both the H and Ps states and the resultant equations are solved separately for each partial wave *L* in momentum space as coupled Lippmann–Schwinger equations. The details are quite involved, and a review of the method has been given recently by Kadyrov and Bray^7^.

In order to extend the calculations above *n*_i_ = 3 a new challenge needs to be met. Given the dominance of the Ps degenerate energy levels on the scattering, larger *n*_i_ will also require the inclusion of larger *l*_i_ ≤ *n*_i_ − 1, and for consistency we also include *l*′ ≤ *n*′ − 1. Due to the state energies (in Rydbergs) being $\varepsilon _{n_{\rm i}}^{{\mathrm{Ps}}} = - 0.5{/}n_{\rm i}^2$ and $\varepsilon _{n{\prime}}^{\mathrm{H}} = - 1{{/}}n\prime^{ 2}$, for Ps(*n*_i_ = 5) we have the desirable (due to the 1/*E* threshold behaviour) exothermic reactions for H(*n*′ ≤ 7). Thus, our calculations require *l*_i_ ≤ 4 and *l*′ ≤ 6. Such large orbital angular momenta generate many more channels in the close-coupling formalism making computation particularly challenging, and our usual approach of using two complete expansions on both centres, impractical. However, following Fabrikant et al.^9^ we take advantage of the fact that the cross-sections of interest are dominated by the interactions involving the degenerate Ps states. Even though we know the importance of the Ps static polarisability coming from its continuum, we can neglect it for our purposes, and only include the *n*_i_ ≤ 5 and *n*′ ≤ 7 eigenstates of the Ps and H Hamiltonians. We tested the validity of this approach by comparing the *n*_i_ ≤ 3 results of the figure with those presented by Rawlins et al.^5^, and found excellent agreement.

### Threshold theory

A Wigner analysis^49^ shows that, if the interaction between reacting particles decays faster than 1/*r* ^2^ at large distances cross-sections for exothermic reactions of the type we are discussing behave near threshold as $E^{l_{{\mathrm{min}}} - 1/2}$, where *l*_min_ is the lowest relative angular momentum *l* in the initial state allowed by symmetry. In our case of Ps and $\bar p$ in the initial state *l* = |*L* − *l*_*i*_|, where *L* is the total angular momentum quantum number. For the cross-section summed over all partial waves *L* we would obtain an *E* ^−1/2^ dependence with the dominance of the *l* = 0 channel. This is the result for Ps(*n*_i_ = 1).

When the long-range effective dipole interaction is present, as for the excited Ps states, it competes with the centrifugal barrier, resulting in modified threshold behaviour^10,12^ which, when the dipole is relatively weak in comparison to the centrifugal barrier, results in partial charge transfer cross-sections, *σ*_*L*_, that behave as *E* ^*λ*−1/2^, where *λ* is a parameter calculated from the effective interaction matrix including both the centrifugal and dipole coupling terms^12^. With the growth of *L* the parameter *λ* grows too and approaches *l*, which can lead to suppression of *σ*_*L*_.

Let us define a boundary value *L*_b_ of the total angular momentum *L* such that for *L* ≤ *L*_b_ the partial cross-section exhibits 1/*E* behaviour, and for *L* > *L*_b_, *E* ^*λ*−1/2^ behaviour. In Table 1 we present *L*_b_ and the threshold exponent *μ* = *λ* − 1/2 for *L* = *L*_b_ + 1. We consider the case of favourable parity, *p* = (−1)^*L*^. For the unfavourable parity *p* = (−1)^*L*+1^ the quantum suppression is stronger. See the main text for further discussion.

### Data availability

All of the presented data, and the full set arising from the discussed Ps-*p* calculations, are available from the authors.
